# The Most Cited Original Articles in Brain Imaging of Children With Cerebral Palsy: A Bibliometric Analysis Between 1984 and 2019

**DOI:** 10.3389/fneur.2020.00955

**Published:** 2020-09-08

**Authors:** Fan Wu, Xiaoyu Wang, Xianjun Li, Haoxiang Jiang, Tingting Huang, Congcong Liu, Miaomiao Wang, Zhonghui Zhai, Xiaoman Zhang, Jingjing Zhang, Heng Liu, Jian Yang

**Affiliations:** ^1^Department of Radiology, The First Affiliated Hospital of Xi'an Jiaotong University, Xi'an, China; ^2^Department of Radiology, Guangzhou Women and Children's Medical Center, Affiliated Guangzhou Medical University, Guangzhou, China; ^3^The Key Laboratory of Biomedical Information Engineering, Ministry of Education, Department of Biomedical Engineering, School of Life Science and Technology, Xi'an Jiaotong University, Xi'an, China; ^4^Department of Radiology, The First Affiliated Hospital of Henan University of TCM, Zhengzhou, China; ^5^Xi'an Jiaotong University Library, Xi'an, China; ^6^Department of Radiology, Medical Imaging Center of Guizhou Province, Affiliated Hospital of Zunyi Medical University, Zunyi, China

**Keywords:** bibliometric analysis, brain imaging, cerebral palsy, children, citation analysis, neuroimaging

## Abstract

**Objective:** Brain imaging is important in diagnosing children with cerebral palsy (CP) and in identifying its etiology. To provide study navigation in this field, a bibliometric analysis was conducted by analyzing the most highly cited articles.

**Methods:** The Web of Science All Databases were used for literature search in this study. All original articles on imaging in children with CP were searched. Two reviewers screened the search results independently and eliminated articles based on exclusion criteria such as participants over 20 years old, topics referring to images outside of the brain, or trauma. According to descending order of yearly citation counts, the top 25% of all included articles were considered as highly cited articles. Information such as yearly citations, research purposes, imaging modalities, CP types, and study designs were recorded and analyzed.

**Results:** A total of 50 highly cited articles ranked by yearly citations (from 23.85 to 3.33, 1991–2018) were included in this study. Considering different research purposes, these studies were classified into three categories: diagnosis studies (*n* = 25; 1991–2017, median: 2011), mechanism studies (*n* = 15; 1999–2018; median: 2014), and prognosis and therapeutic effect studies (*n* = 10; 2008–2017; median: 2014.5). First, for diagnosis studies, 22 studies used single modality and three used multi-modalities; the majority of these studies focused on diagnostic value evaluation (*n* = 10) and image performance (*n* = 12) of a single type of CP (*n* = 15) by using descriptive (*n* = 14) or cross-sectional approaches (*n* = 10). Second, for mechanism studies, the ratio between single and multi-modality was 8:7; most of these studies concentrated on a single subtype of spastic CP (hemiplegia = 10, quadriplegia = 2) with a cross-sectional study design (*n* = 10). Third, regarding the prognosis and therapeutic effect studies, the single vs. multi-modality ratio was 5:5, and these studies were dedicated to the efficiency of constraint-induced movement therapy in children with hemiplegia; paired design trials (*n* = 6) and randomized controlled trials (*n* = 2) were used more frequently.

**Conclusion:** Studies using multi-modality and high-level evidence-based design to provide information regarding mechanism, prognosis, and therapeutic efficacy may be the potential future research direction in the field of CP research.

## Introduction

Cerebral palsy (CP) is a group of permanent movement disorders attributed to non-progressive disturbances occurring during fetal or infant brain development (1). The prevalence of CP for all live birth ranges from 2 to 3 per 1,000 live births (2). However, this prevalence varies for different regions or countries depending on income level, and a higher prevalence has also been reported in infancy compared to all live births (1, 2). Brain sonography, a diagnostic imaging tool, was the first technique used to detect brain lesions in CP patients at 1984 (3). Moreover, in 2004, the American Academy of Neurology and the Child Neurology Society jointly published a practice parameter recommending neuroimaging examination as a necessary evaluation procedure for CP children with uncertain etiology (4). In recent years, the field of neuroimaging has made significant progress in early and accurate CP diagnosis (5), mechanism of action, prediction of therapy effect, and prognosis (functionality) (6–8). Brain imaging has become one of the main focuses of CP research, with numerous articles published on this topic. In the current imaging evaluation era of CP, a substantial volume of literature comprising studies on varying topics and designs are available to neurologists, pediatric neurologists, pediatricians, and radiologists. However, due to the lack of quantitative research providing an overview of brain imaging studies in CP patients (9), it is challenging for students, residents, and researchers new to the field to identify the most important study areas and future direction. Therefore, distilling the newest and emerging research in this field through citation counts can help create an evidence-based approach to guide future study.

Bibliometric analysis was previously used to quantitatively survey knowledge development in imaging and neuroimaging fields (10). Compared to meta-analysis and systemic review, which both focus on specific questions such as study population, methods, and findings, the bibliometric analysis provides an overview of the study field from a different angle, by using bibliographic material such as yearly citations, authors' information, and impact factor (IF) of the publishing journals (11). These three types of studies have distinct focuses and provide information that can complement each other in a given field. According to our research, no previous bibliometric analysis has been done on brain imaging of children with CP. Thus, we have selected the bibliometric analysis method to quantitatively evaluate the knowledge structure and development of pediatric CP imaging, to highlight emerging themes and future study trends in this field.

This study aimed to perform a citation analysis of the most cited papers on brain imaging in children with CP and analyzed each paper individually according to imaging modality, year of publication, yearly citations, research purposes, study designs, country of origin of the first author, and IF of the journal.

## Materials and Methods

As our study was a retrospective bibliometric analysis of publicly available study literature, it was exempt from institutional review board approval.

### Literature Search

To identify the most highly cited articles, we conducted a systematic literature search on CP brain imaging for children articles, published from November 4, 2019. We used the Web of Science All Databases (Clarivate Analytics, Philadelphia, United States), which included the MEDLINE database; the results were limited to the English language with no publication year limit. The searching topics were listed:

“cerebral palsy” or “brain paralysis” or “monoplegia” or “unilateral paralysis” or “diplegia” or “quadriplegia” or “tetraplegia” or “hemiplegia” or “hemiparesis” or “static encephalopathy”

and

“diffusion tensor imaging” or “DTI” or “diffusion kurtosis imaging” or “DKI” “neuroradiology” or “neuroimaging” or “brain imaging” or “brain CT” or “head CT” or “computed tomography” or “MRI” or “MR imaging” or “magnetic resonance imaging” or “DWI” or “diffusion-weighted imaging” or “MR perfusion” or “magnetic resonance perfusion” or “SPECT” or “PET” or “sonography” or “ultrasound” or “doppler” or “network” or “connect^*^” and “infant^*^” or “child^*^” or “pediatric” or “toddler^*^” or “bab^*^” or “trottie” or “kid^*^” or “neonate^*^” or “newborn^*^” or “adolescent^*^” or “teenager” or “juvenile^*^” or “teen^*^”.

All original articles on imaging in children with CP were included. Exclusion criteria were articles concerning participants over 20 years old, or topics referring to images of the spinal cord, musculoskeletal, urinary systems, vascular ultrasounds, ultrasound-guided therapy, hemi-convulsion-hemiplegia syndromes, or trauma.

### Screening of Highly Cited Original Articles

Two reviewers (Fan Wu and Jingjing Zhang) screened the search results independently. Full-text articles were retrieved and screened according to the inclusion and exclusion criteria. In case of doubt, a third reviewer (Heng Liu) participated in the screening. Two reviewers independently extracted data and stored them electronically in Microsoft Excel 2016. The results were downloaded to a local database. From a list of articles in descending order of yearly citations (total citation count divided by the year difference between publication year and 2019), the top 25% were considered the most highly cited (12).

### Data Analysis

For the most highly cited articles in the final analysis, the following information was collected and listed: yearly citations, total citations, research purposes, CP types, study designs, imaging modalities, subjects age, modality of study acquisition, country of the first author, publication journal, and journal IF. For descriptive analysis of the CP types, imaging modalities, study designs, author or publication information, and number and proportion of articles were listed.

## Results

Among 6,137 published articles, 202 on brain imaging in children with CP were identified based on our inclusion and exclusion criteria. A total of 50 articles were defined as the most highly cited articles according to the ranking number of yearly citations.

### Top 50 Highly Cited Original Articles

Table 1 lists the bibliometric materials of the top 50 highly cited original articles, regarding particulars such as citation counts, imaging modalities, and author and publication information. The yearly citation counts ranged from 23.85 to 3.33 per year (median: 6/year), and the total citation counts were between 310 and 7 (median: 45.5 times). According to different research purposes, the articles were classified into three categories: CP diagnosis studies (25 articles, 50%), CP mechanism studies (15 articles, 30%), and CP prognosis and therapeutic effect studies (10 articles, 20%). Based on the above classification, we explored the imaging modalities, CP types, impaired functions, study designs, country of origin of the first author, and journal's IF of these articles.

**Table 1:** The top 50 highly cited brain imaging articles in children with cerebral palsy ranked by yearly average citation.

**Rank**	**Article name**	**Main author**	**Journal**	**IF (2018)**	**Brain imaging modality**	**Single modality; multiple modality**	**Age**	**Modality of study acquisition**	**Publication Year**	**No. of yearly citations**	**No. of total citations**	**Rank of total citations**
1	Clinical and MRI correlates of cerebral palsy—the European cerebral palsy study	Bax, M.	*JAMA*	51.27	sMRI	Single	0.08–7.25 years	-	2006	23.85	310	1
2	Sensory and motor deficits in children with cerebral palsy born preterm correlate with diffusion tensor imaging abnormalities in thalamocortical pathways	Hoon, A. H. Jr.	*Developmental Medicine and Child Neurology*	3.53	DTI	Single	1.25–15 years	Sedation	2009	17.4	174	3
3	Quantitative diffusion tensor imaging in cerebral palsy due to periventricular white matter injury	Thomas, B.	*Brain*	11.81	DTI	Single	12–16 years	Awake	2005	13.21	185	2
4	Effect of autologous cord blood infusion on motor function and brain connectivity in young children with cerebral palsy: a randomized, placebo-controlled trial	Sun, J. M.	*STEM CELLS Translational Medicine*	5.96	DTI, sMRI, and DWI	Multiple	1–6 years	–	2017	12	24	37
5	Corticospinal tract diffusion properties and robotic visually guided reaching in children with hemiparetic cerebral palsy	Kuczynski, A. M.	*Human Brain Mapping*	4.55	DTI	Single	6–19 years	–	2018	11	11	48
6	Hand function in relation to brain lesions and corticomotor-projection pattern in children with unilateral cerebral palsy	Holmstrom, L.	*Developmental Medicine and Child Neurology*	3.53	sMRI and TMS	Multiple	7–16 years	–	2010	10.22	92	10
7	Function and neuroimaging in cerebral palsy: a population-based study	Himmelmann, K.	*Developmental Medicine and Child Neurology*	3.53	sMRI or CT	Single	4–8 years	–	2011	9.5	76	15
7	MRI classification system (MRICS) for children with cerebral palsy: development, reliability, and recommendations	Himmelmann, K.	*Developmental Medicine and Child Neurology*	3.53	sMRI	Single	–	–	2017	9.5	19	43
9	Quantitative diffusion tensor tractography of the motor and sensory tract in children with cerebral palsy	Yoshida, S.	*Developmental Medicine and Child Neurology*	3.53	DTI	Single	0.33–9 years	Partial sedation	2010	8.78	79	14
10	Diffusion tensor imaging in children with periventricular leukomalacia: variability of injuries to white matter tracts	Nagae, L. M.	*American Journal of Neuroradiology*	3.26	DTI	Single	1.33–13.25 years	Sedation	2007	8.75	105	8
11	Cerebral palsy in a term population: risk factors and neuroimaging findings	Wu, Y. W.	*Pediatrics*	5.4	sMRI or CT	Single	–	–	2006	8.62	112	5
12	Diffusion tensor imaging of periventricular leukomalacia shows affected sensory cortex white matter pathways	Hoon, A. H.	*Neurology*	8.69	DTI	Single	4–8 years	–	2002	8.47	144	4
13	Structural neuroplastic change after constraint-induced movement therapy in children with cerebral palsy.	Sterling, C.	*Pediatrics*	5.4	MRI	Single	2.08–7.50 years	Sedation	2013	7.83	47	22
14	Is outcome of constraint-induced movement therapy in unilateral cerebral palsy dependent on corticomotor projection pattern and brain lesion characteristics?	Islam, M.	*Developmental Medicine and Child Neurology*	3.53	sMRI and TMS	Multiple	8–16 years	–	2014	7.8	39	28
15	Cortical somatosensory reorganization in children with spastic cerebral palsy: a multimodal neuroimaging study	Papadelis, C.	*Frontiers in Human Neuroscience*	2.87	DTI, fMRI, and MEG	Multiple	4.75–17 years	Awake	2014	7.8	39	28
16	Assessment of the structural brain network reveals altered connectivity in children with unilateral cerebral palsy due to periventricular white matter lesions.	Pannek, K.	*NeuroImage-Clinical*	3.94	sMRI and DTI	Multiple	5–17 years	–	2014	7.4	37	30
16	Magnetic resonance imaging findings in a population-based cohort of children with cerebral palsy	Robinson, M. N.	*Developmental Medicine and Child Neurology*	3.53	sMRI	Single	5–7 years	–	2009	7.4	74	16
18	MRI structural connectivity, disruption of primary sensorimotor pathways, and hand function in cerebral palsy.	Rose, S.	*Brain Connectivity*	–	sMRI and DTI	Multiple	10.63 ± 3.00 years	–	2011	7.38	59	18
19	Population-based study of neuroimaging findings in children with cerebral palsy	Towsley, K.	*European journal of Pediatric Neurology*	2.5	sMRI or CT	Single	0–5.50 years	–	2011	7.25	58	20
20	Reorganization of the somatosensory cortex in hemiplegic cerebral palsy associated with impaired sensory tracts.	Papadelis, C.	*NeuroImage-Clinical*	3.94	MEG, sMRI, and DTI	Multiple	6–17 years	–	2018	7	7	49
21	Speech problems affect more than one in two children with cerebral palsy: Swedish population-based study	Nordberg, A.	*Acta Paediatrica*	2.27	sMRI or CT	Single	–	–	2013	6.67	40	26
22	Quantitative analysis of brain pathology based on MRI and brain atlases- applications for cerebral palsy	Faria, A. V.	*Neuroimage*	5.81	DTI	Single	4–13 years	Sedation	2011	6.5	52	21
23	Brain structural connectivity increases concurrent with functional improvement: evidence from diffusion tensor MRI in children with cerebral palsy during therapy	Englander, Z. A.	*NeuroImage-Clinical*	3.94	DTI	Single	1.10–5.10 years	–	2015	6.25	25	36
24	Sensory tractography and robot-quantified proprioception in hemiparetic children with perinatal stroke	Kuczynski, A. M.	*Human Brain Mapping*	4.55	DTI	Single	6–19 years	–	2017	6	12	46
24	Using diffusion tensor imaging to identify corticospinal tract projection patterns in children with unilateral spastic cerebral palsy.	Kuo, H. C.	*Developmental Medicine and Child Neurology*	3.53	DTI and TMS	Multiple	6.08–17.08 years	–	2017	6	12	46
24	Validity of semi-quantitative scale for brain MRI in unilateral cerebral palsy due to periventricular white matter lesions: relationship with hand sensorimotor function and structural connectivity	Fiori, S.	*NeuroImage-Clinical*	3.94	sMRI and DTI	Multiple	11.40 ± 3.10 years	–	2015	6	24	37
27	Neuroradiology can predict the development of hand function in children with unilateral cerebral palsy	Holmefur, M.	*Neurorehabilitation and Neural Repair*	3.76	sMRI or CT	Single	0.75–9 years	–	2013	5.83	35	31
28	Gastrointestinal manifestations in children with cerebral palsy.	Del, G. E.	*Brain & Development*	1.76	sMRI or CT	Single	0.50–12 years	–	1999	5.6	112	5
29	Capturing neuroplastic changes after bimanual intensive rehabilitation in children with unilateral spastic cerebral palsy: a combined DTI, TMS and fMRI pilot study	Bleyenheuft, Y.	*Research in Developmental Disabilities*	1.87	DTI, fMRI, and TMS	Multiple	6–9 years	–	2015	5.5	22	40
30	DTI-based three-dimensional tractography detects differences in the pyramidal tracts of infants and children with congenital hemiparesis	Glenn, O. A.	*Journal of Magnetic Resonance Imaging*	3.73	DTI	Single	0.83–3.67 years	–	2003	5.44	87	11
31	Diffusion tensor imaging study of the response to constraint-induced movement therapy of children with hemiparetic cerebral palsy and adults with chronic stroke	Rickards, T.	*Archives of Physical Medicine and Rehabilitation*	2.7	DTI	Single	2.10–7.60 years	Sedation	2014	5.4	27	34
31	Reliability of a novel, semi-quantitative scale for classification of structural brain magnetic resonance imaging in children with cerebral palsy	Fiori, S.	*Developmental Medicine and Child Neurology*	3.53	sMRI	Single	4–16.92 years	–	2014	5.4	27	34
33	Correlation of quantitative sensorimotor tractography with clinical grade of cerebral palsy	Trivedi, R.	*Neuroradiology*	2.5	DTI	Single	3–12 years	–	2010	5.11	46	24
34	Diffusion tensor MR imaging tractography of the pyramidal tracts correlates with clinical motor function in children with congenital hemiparesis	Glenn, O. A.	*American journal of Neuroradiology*	3.26	DTI	Single	0.54–17.44 years	–	2007	4.92	59	18
35	Neuroplastic sensorimotor resting state network reorganization in children with hemiplegic cerebral palsy treated with constraint-induced movement therapy.	Manning, K. Y.	*Journal of Child Neurology*	2.09	sMRI and fMRI	Multiple	6–18 years	Awake	2016	4.67	14	45
36	An Australian population study of factors associated with MRI patterns in cerebral palsy	Reid, S. M.	*Developmental Medicine and Child Neurology*	3.53	sMRI	Single	0.08–11 years	–	2014	4.4	22	41
37	MRI and clinical characteristics of children with hemiplegic cerebral palsy	Cioni, G.	*Neuropediatrics*	1.65	sMRI	Single	1–18.3 years	–	1999	4.25	85	12
38	Periventricular leukomalacia: Relationship between lateral ventricular volume on brain MR images and severity of cognitive and motor impairment	Melhem, E. R.	*Radiology*	7.61	sMRI	Single	1.50–12.50 years	–	2000	4.21	80	13
39	Autosomal recessive spastic tetraplegia caused by AP4M1 and AP4B1 gene mutation: expansion of the facial and neuroimaging features	Tuysuz, B.	*American journal of Medical Genetics Part A*	3.26	sMRI	Single	2.50–17 years	–	2014	4.2	21	42
40	Correlation between the degree of periventricular leukomalacia diagnosed using cranial ultrasound and MRI later in infancy in children with cerebral-palsy	Devries, L. S.	*Neuropediatrics*	1.65	sMRI and TCS	Multiple	0.92–2.67 years	Sedation	1993	4.19	109	7
41	Treatment-induced plasticity in cerebral palsy: a diffusion tensor imaging study	Trivedi, R.	*Pediatric Neurology*	2.33	DTI	Single	3–12 years	Sedation	2008	4.09	45	25
42	Diffusion tensor imaging demonstrates focal lesions of the corticospinal tract in hemiparetic patients with cerebral palsy	Son, S. M.	*Neuroscience Letters*	2.17	DTI	Single	0.90–7 years	–	2007	3.92	47	22
43	Diffusion MRI in corticofugal fibers correlates with hand function in unilateral cerebral palsy	Holmstrom, L.	*Neurology*	8.69	DTI	Single	7.20–17.30 years	–	2011	3.75	30	32
43	Resting state and diffusion neuroimaging predictors of clinical improvements following constraint-induced movement therapy in children with hemiplegic cerebral palsy.	Manning, K. Y.	*Journal of Child Neurology*	2.09	DTI and fMRI	Multiple	6–15 years	Awake	2015	3.75	15	44
45	Magnetic-resonance-imaging in children with spastic diplegia—correlation with the severity of their motor and mental abnormality	Yokochi, K.	*Developmental Medicine and Child Neurology*	3.53	sMRI	Single	3–10 years	–	1991	3.71	104	9
46	Athetotic and spastic cerebral palsy: Anatomic characterization based on diffusion-tensor imaging	Yoshida, S.	*Radiology*	7.61	DTI	Single	0.5–15 years	Partial sedation	2011	3.5	28	33
46	Effect of sensory and motor connectivity on hand function in pediatric hemiplegia	Gupta, D.	*Annals of Neurology*	9.5	sMRI, DTI, and TMS	Multiple	7.02–18.12 years	–	2017	3.5	7	49
48	Diffusion tensor imaging demonstrated radiologic differences between diplegic and quadriplegic cerebral palsy.	Chang, M. C.	*Neuroscience Letters*	2.17	DTI	Single	0.10–5.30 years	–	2012	3.43	24	37
49	MRI findings in patients with spastic cerebral palsy.1. Correlation with gestational age at birth	Okumura, A.	*Developmental Medicine and Child Neurology*	3.53	sMRI	Single	1–19 years	–	1997	3.36	74	16
50	Corticospinal dysgenesis and upper-limb deficits in congenital hemiplegia: a diffusion tensor imaging study	Bleyenheuft, Y.	*Pediatrics*	5.4	sMRI and DTI	Multiple	10–16 years	–	2007	3.33	40	26

*TCS, transcranial ultrasound; sMRI, structural magnetic resonance imaging; DTI, diffusion tensor imaging; CT, computed tomography; fMRI, functional magnetic resonance imaging; TMS, transcranial magnetic stimulation; MEG, magnetoencephalography; DWI, diffusion-weighted imaging*.

### Application of Imaging Modalities in the Top 50 Highly Cited Articles

**Figure 1:**
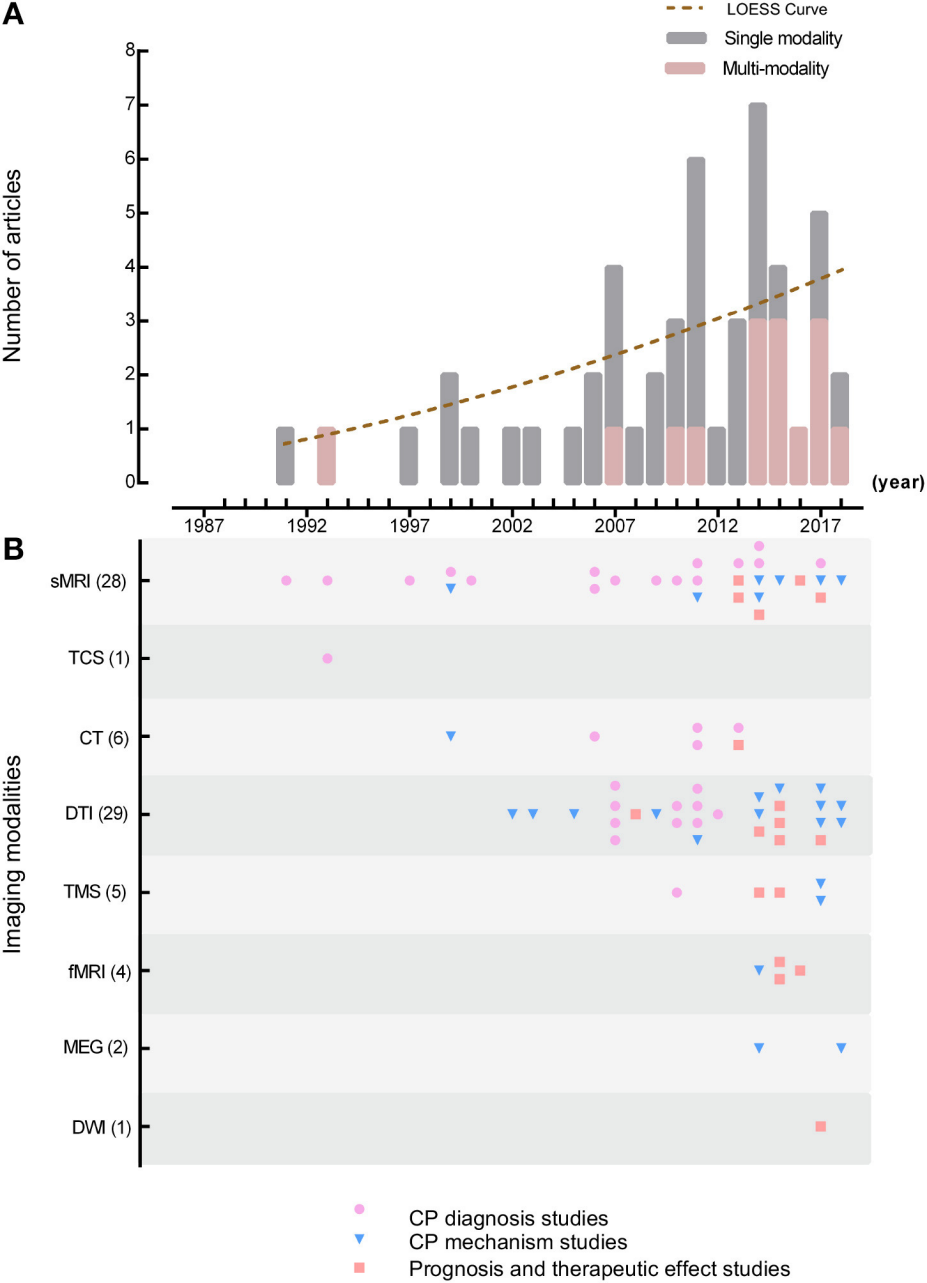
Distribution of imaging modality and study topics. **(A)** Yearly article counts distribution of the top 50 highly cited original articles utilized single or multiple modality; **(B)** yearly article counts for each imaging modality used in the top 50 highly cited original articles classified by different research purposes. Different research purposes included CP diagnosis studies, CP mechanism studies, and CP prognosis and therapeutic affect studies. Study purposes are presented in different colors. sMRI, structural magnetic resonance imaging; TCS, transcranial ultrasound; CT, computed tomography; DTI, diffusion tensor imaging; TMS, transcranial magnetic stimulation; fMRI, functional magnetic resonance imaging; MEG, magnetoencephalography; DWI, diffusion-weighted imaging.

Figure 1A shows the yearly publication count distribution of the top 50 highly cited original articles that applied single or multiple modalities. The article publication numbers showed an increasing trend over time. Thirty-five studies used single modality (CP diagnosis: *n* = 22; CP mechanism: *n* = 8; CP prognosis and therapeutic effect: *n* = 5), whereas 15 studies were multi-modality studies with a growth trend in the past 10 years (CP diagnosis: *n* = 3; CP mechanism: *n* = 7; CP prognosis and therapeutic effect: *n* = 5). Among multi-modality studies, multiple imaging modalities were applied in eight studies. Specifically, seven studies used two imaging modalities [structural magnetic resonance imaging (sMRI) (1991) and transcranial ultrasound (TCS) = 1, 1993; sMRI and diffusion tensor imaging (DTI) = 4, 2007–2015; DTI and functional MRI (fMRI) = 1, 2015; sMRI and fMRI = 1, 2016], and one study used three imaging modalities [DTI, sMRI, and diffusion-weighted imaging (DWI) = 1, 2017]. Seven studies combined an imaging modality with an electrophysiological modality [magnetoencephalography (MEG) = 2, 2014 and 2018; transcranial magnetic stimulation (TMS) = 5, 2010–2017]. Thus, combining an imaging modality with an electrophysiological modality could be a possible study direction for brain imaging studies in children with CP.

Figure 1B shows the yearly article counts of each image modality used in the top 50 highly cited original articles according to different research purposes. First, sMRI (1991) and TCS (1992) were used the earliest in CP diagnosis studies (*n* = 25), and DTI (29 times) and sMRI (29 times) were used the most. Second, computed tomography (CT) was applied as a complementary method to sMRI in six CP diagnosis studies to help identify imaging performance. Third, multi-modalities were applied more frequently in CP mechanism studies since 2011 (8/15 studies), especially DTI combined with other MR technologies, TMS and MEG. Last, the prognosis (*n* = 1) and therapeutic affect studies (*n* = 9) first appeared in 2008, and the utilization of multi-modality in these types of studies has increased remarkably from 2013 (*n* = 5).

### CP Types, Impaired Functions, and Study Designs of the Top 50 Highly Cited Articles

Table 2 lists the article counts of CP types, impaired functions, and study designs of the top 50 highly cited articles, as well as their publication years and distribution among different research purposes. CP diagnosis studies were further classified into diagnostic value of imaging methods (1993–2014, median: 2007.5), brain image performance (1991–2012, median: 2008.5), imaging classification systems, and imaging post-processing applications (2011–2017, median: 2014). The CP mechanism studies (1999–2018, median: 2014) have been explored from the 1990's. The prognosis and therapeutic effect studies involved four distinct types of treatment (2008–2017, median: 2014.5) presenting with a number of increases in the past 10 years.

**Table 2:** Cerebral palsy types, impaired functions, and study designs of the top 50 highly cited articles in different research purposes.

				**CP diagnosis studies**			**CP mechanism studies**	**CP prognosis and therapeutic effect studies**					**Published year (median)**	**Total**
				**Diagnostic value of imaging method**	**Image performance**	**Imaging classification system and applications in CP**		**Autologous cord blood infusion**	**Constraint-induced movement therapy**	**Bimanual intensive rehabilitation**	**Botulinum injection**	**Prediction of function development**		
CP types	Spastic CP	Single subtype:	Hemiplegia	1	5		10		5	1		1	1999–2018 (2014)	23
			Diplegia		2*								1991, 2012	2
			Quadriplegia		2*		2				1		2008–2014 (2010)	5
		Mixed subtypes:			1	2	1	1					2010–2015 (2014)	5
		Unclassified subtypes		1	2^†^								1997–2011 (2000)	3
	Athetoid CP				1^†^								2011	1
	Unclassified or multiple CP type mixed			8	1	1	2	1					1993–2017 (2009)	13
Impaired functions	Sensorimotor function			1	5*¶		3	1	2^‡^		1		1991–2017 (2008)	13
	Specific motor function		Hand or upper limb function-related	1#	4^†^		8		4^‡^	1		1	1999–2018 (2014)	19
		Lower limb function-related		1#	1^†^								1999, 2012	2
	Cognitive and function (including speech)			2#	2*¶								1991–2013 (1999.5)	4
Studies not related to impaired function				7	3	3	4	1					1993–2017 (2010.5)	18
Study designs	Descriptive study			10	2	2	5						1999–2017 (2011)	19
	Cross-sectional study				9	1	10						2000–2018 (2010.5)	20
	Case–control study				1								2007	1
	Cohort study											1	2013	1
	Non-randomized controlled trial								1				2016	1
	Paired design trial								4	1	1		2008–2015 (2014)	6
	Randomized controlled trial							2					2015, 2017	2
Published year (median)				1993–2014 (2007.5)	1991–2012 (2008.5)	2011–2017 (2014)	1999–2018 (2014)	2015, 2017	2013–2016 (2014)	2015	2008	2013	1991–2018 (2011)	
				1991–2017 (2011)				2008–2017 (2014.5)						
Total articles				10	12	3	15	2	5	1	1	1	50	

The *, #, ¶, and ‡ symbols signify repeated counting of one article.

#### CP Types and Impaired Functions

Regarding the CP types, articles mainly focused on single CP type study, specifically spastic CP (SCP) (37/50, 74%) (Table 2). The single subtype studies constituted of approximately 78% (29/37) spastic CP studies, and hemiplegia constituted of 79% (23/29) single subtype studies. Limited to early imaging technological development, most studies concentrated on the diagnostic value of specific imaging methods in CP (8/10) or identified unique image performances and feature classification in different CP types or subtypes (13/15). The majority of CP mechanism studies focused on single subtype SCP, particularly hemiplegia (10/15) and quadriplegia (2/15). The prognosis and therapeutic effect studies concentrated closely on constraint-induced movement therapy (CIMT) (5/10), bimanual intensive rehabilitation (1/10), and hand function prediction (1/10) of children with hemiplegia. Moreover, an article on botulinum injection studied patients with quadriplegia (1/10), and the autologous cord blood infusion studies (2/10) were also performed in children with CP but did not mention any particular CP subtype.

Concerning impaired functions, 32 out of 50 articles included impaired function in their studies (64%). Hand or upper limb function (19/50, 38%) and sensorimotor pathway function (13/50, 26%) received the most attention from researchers. In addition, four articles described the relationship between brain structure changes and cognitive functions (including speech function, 1991–2014). For different research purposes, the CP diagnosis studies focused on the correlation between brain image performance and impaired functions (12/25). CP mechanism studies were mostly concerned with the process of hand or upper limb and sensorimotor function (11/15). While therapeutic effect studies used CIMT (5/10), bimanual intensive rehabilitation (1/10), autologous cord blood infusion (1/10), and botulinum injection (1/10), the assessment of hand or upper limb function (5/10) or sensorimotor function (4/10) were also involved. However, only one study in development outcome prediction paid attention to hand function in hemiplegia.

#### Study Designs

Among the top 50 highly cited articles, 78% (39/50) were descriptive (19/50, 38%) and cross-sectional studies (20/50, 40%) (Table 2). The majority of the CP diagnosis and mechanism studies followed these two study designs. Furthermore, prospective design was adopted in a cohort study of prognosis (1/50, 2%) and in therapeutic effect evaluation studies (9/50, 18%). Specifically, therapeutic effect evaluation studies included six paired design trials (12%), two randomized controlled trials (4%), and one non-randomized controlled trial (2%).

Considering that the age range of children at the time of neuroimaging might affect the choice of study design, these subjects were divided into pre- (0–3 years) and post-myelination period (>3 years). Only one study included children at the pre-myelination period (11–32 months); 23 studies included children at the post-myelination period (23/50, 46%); 23 studies included children at both the pre- and post-myelination period, while 3 studies did not mention the children's age range.

Thirteen studies mentioned information on the modality of study acquisition (awake, natural sleep, sedation, and general anesthesia). Four studies reported that children were awake during the scanning (one article was published in 2005, ages: 12–16 years; three articles were published between 2014 and 2016, ages: 4.75–18 years); two mentioned that children were sedated at the age of 5 years or younger or have difficulty remaining still, while other children over 5 years were screened while awake (published between 2010 and 2011, ages: 0.33–15 years). Seven studies reported that all children included were sedated for the neuroimaging examination (published between 1993 and 2014, ages: 0.92–15 years), with five of them describing specific sedative drugs and doses in detail. From the above we can tell that only a few articles reported information on the modality of study acquisition. In articles including modality information of the study acquisition, the transition of obtaining images from sedated children to awake children can be observed. Thus, we believe that more attention needs to be paid on the safe acquisition of imaging data of children with CP.

### Authors and Publications of the Top 50 Highly Cited Articles

#### First Author Countries

The country of affiliation of the first author is listed in descending order: USA (16/50, 32%), Sweden (7/50, 14%), Canada (5/50, 10%), Italy (4/50, 8%), Australia (4/50, 8%), Japan (4/50, 8%), Belgium (3/50, 6%), India (2/50, 4%), South Korea (2/50, 4%), England (1/50, 2%), Netherlands (1/50, 2%), and Turkey (1/50, 2%).

#### Journals

The top 50 highly cited articles were published in 27 journals. The top three journals with the most published articles on pediatric CP were *Developmental Medicine and Child Neurology* (12 articles, 24%), *NeuroImage: Clinical* (4 articles, 8%), and *Pediatrics* (3 articles, 6%). A full list of the article number and most recent journal IFs is shown in Table 3. The journals with the top three IFs in 2018 were *JAMA* (one article, 2%), *Brain* (one article, 2%), and *Annals of Neurology* (one article, 2%).

**Table 3:** The impact factors and rank of journals which published articles on pediatric cerebral palsy.

**Journal**	**No. of articles (%)**	**IF (2018)**	Rank^*^
*Developmental Medicine and Child Neurology*	12 (24)	3.53	1
*NeuroImage: Clinical*	4 (8)	3.94	2
*Pediatrics*	3 (6)	5.40	3
*Neurology*	2 (4)	8.69	4
*Radiology*	2 (4)	7.61	5
*Human Brain Mapping*	2 (4)	4.55	6
*American Journal of Neuroradiology*	2 (4)	3.26	7
*Neuroscience Letters*	2 (4)	2.17	8
*Journal of Child Neurology*	2 (4)	2.09	9
*Neuropediatrics*	2 (4)	1.65	10
*JAMA*	1 (2)	51.27	11
*Brain*	1 (2)	11.81	12
*Annals of Neurology*	1 (2)	9.50	13
*STEM CELLS Translational Medicine*	1 (2)	5.96	14
*Neuroimage*	1 (2)	5.81	15
*Neurorehabilitation and Neural Repair*	1 (2)	3.76	16
*Journal of Magnetic Resonance Imaging*	1 (2)	3.73	17
*Frontiers in Human Neuroscience*	1 (2)	2.87	18
*Archives of Physical Medicine and Rehabilitation*	1 (2)	2.70	19
*Neuroradiology*	1 (2)	2.50	20
*European Journal of Pediatric Neurology*	1 (2)	2.50	21
*Pediatric Neurology*	1 (2)	2.33	22
*Acta Paediatrica*	1 (2)	2.27	23
*American Journal of Medical Genetics Part A*	1 (2)	2.20	24
*Research in Developmental Disabilities*	1 (2)	1.87	25
*Brain & Development*	1 (2)	1.76	26
*Brain Connectivity*	1 (2)	–	27

*IF data were collected from Journal Citation Reports for the year 2018. No., number; IF, impact factor; JAMA, Journal of the American Medical Association*.

* *The journals ranked by number of articles and IF*.

## Discussion

Targeting the unclear focus of current studies and future directions in brain imaging for children with CP, our study demonstrated that the main topics of the highly cited articles were the identification and diagnosis of CP (44%) and understanding its mechanism using MR technologies (30%), with designs based on low-level evidence. Our results have also indicated that possible future directions in this field could be research on therapy effect evaluation and prognosis using multi-modalities, based on high-level evidence design (2008–2017, median: 2014.5). Moreover, CP studies on the single subtype SCP and sensorimotor or upper limb-related function have also attracted considerable attention.

This study analyzed the bibliometric information of the top 25% of articles in order of yearly citation counts in brain imaging for children with CP, since brain imaging has first been applied in CP studies in 1984 (3). According to our research, there is still a lack of agreement on the standard of highly cited articles worldwide. Levitt et al. study mentioned that they chose the top 25% of yearly citation counts as highly cited articles due to it covering enough articles in the field while still meeting the definition of highly cited articles (12). Therefore, we chose the same standard, namely, the top 25%. Figure 1 shows that no articles before 1991 were included, although no time limit was set for this study. According to the search results, only three articles were found before 1991, with yearly citation counts between 0.20 and 1.93, which did not qualify these articles for the top 25% of the list.

The selection of imaging techniques in CP studies changed from subjective and descriptive stages to objective and quantitative stages. Before the 21st century, TCS, sMRI, and CT were the first few technologies used in CP studies to help identify brain abnormalities and etiology (13). In the 2000's, with the development of imaging technology, DTI became the most frequently applied method to quantitatively analyze structural changes, mechanisms, and therapy effectiveness in CP children; it provided objective results for the detection of brain alterations (14, 15). From 2010 to 2018, the multi-modality approaches were more frequently used to assess therapy effectiveness and comprehensively discover mechanisms in children with CP (16–18). This was the result of a high clinical need for the discovery of the relationship between structure and function, in order to improve treatment evaluation (19, 20). The practice parameters from the American Academy of Neurology and the Child Neurology Society also recommend the use of MRI over CT in children with CP, due to its higher rate in identifying etiology and timing (4). Therefore, the application of new MR technologies became mainstream in brain imaging studies of children with CP. The historical trend in brain imaging modalities also reflects the rapid application of the new imaging techniques applied in CP studies. From the identification of lesions to the exploration of fiber pathways and the construction of brain networks, neuroimaging combined with TMS and/or MEG assisting the comprehensive evaluation of etiology, therapy effectiveness, and prognosis with multi-modality has become the route for future studies.

Among the CP diagnosis studies, the earlier descriptive studies have shown the diagnostic value of imaging examination in identifying the etiology for CP patients (21, 22). Meanwhile, the studies on image performance and features appeared to help understand the causes of CP and identify the relationship between brain abnormalities and clinical characteristics (13, 23). With the development of MR quantitative technology, studies mainly focused on the relevant relationship between structural changes and various functions including sensorimotor function, cognitive (including speech) function, and upper or lower limb function (14, 24). Recently, study focus has been shifted from etiology identification to systematically summarizing and classifying the correlation of brain abnormality with possible etiology (25, 26). The time of CP diagnosis has also been moved forward to 6 months of corrected age using MRI with a predictive sensitivity of 80–90% (5). Furthermore, new evidence suggests that 14% of CP cases have a genetic component (5). Utilizing molecular genetic techniques combined with neuroimaging to detect specific brain abnormalities has become a new tendency in CP studies (27).

CP studies have used new imaging techniques and multi-modality to explore CP mechanisms (19, 20). These studies concentrated mostly on CP subtypes, particularly hemiplegia, due to its extremely unique pathological mechanism (28). Congenital hemiparesis has become an ideal study model disorder due to its abnormal motor function only present on one side of the body, with the contralateral normal side serving as an internal control (29). Considering the significance of the upper limb and hand function for self-care ability in daily life, most of the studies deeply explored the changes in center-hand pathways and structural neuroplasticity (19, 20). Some studies also focused on sensorimotor function-related changes (15). However, very few studies have been found on the mechanism of impaired cognitive functions in children with CP, which needs to be explored in the future. Despite the high incidence of reports on SCP, further investigation is still needed on the mechanisms of other CP subtypes, and an evidence-based study is also essential.

Brain imaging was first implicated in therapeutic evaluation of children with CP in 2008 (30) among 50 highly cited articles. Since then, use of brain imaging to evaluate therapeutic effect in children with CP has been widely adopted. According to our study, nine articles on the evaluation of therapeutic effect were found after 2008, and seven articles have been published in the last 5 years. The utilization of imaging modality changed from single modality evaluation (30, 31) to multi-modality evaluation combined with a functional modality such as TMS (32). The treatments evaluated have also changed from single treatment efficacy evaluation (30, 31) to multiple treatment efficacy evaluation, accompanied by the development of possible treatment-specific biomarkers (33). Thus, we can conclude that the use of brain imaging techniques to evaluate therapeutic effect has become the current research focus and can lead the direction of future study.

The continental distribution of the first author includes North America, Europe, Australia, and Asia. The distribution is uneven with a trend toward developed countries. However, in developing countries, more attention is needed in the medical treatments of high-risk CP populations. Especially due to the financial constraints of low-level economies, the medical and social security systems are underdeveloped, which further affect quality of life of children with CP.

The journals *Developmental Medicine and Child Neurology, NeuroImage: Clinical*, and *Pediatrics* featured prominently among the top 50 journals, highlighting their important contribution in shaping the diagnosis, mechanism, and treatment of CP. Several well-known high-IF journals were included in the top 50, for example *JAMA, Brain*, and *Annals of Neurology*. It is worth mentioning that *JAMA* published the most cited article in 2006. Although its original publication was 10 years ago, it still maintains the highest number of citations per year. It should also be noted that *STEM CELLS Translational Medicine* and *NeuroImage* have published two articles representing randomized clinical trials on autologous cord blood infusion.

### Limitations

There are several limitations to this study. First, we searched the Web of Science All Databases to consider authority and relative comprehensiveness. The literature found in other databases and not included in the Web of Science All Databases was not highly cited. Second, this analysis contained all the original articles. The longer the period since the article was published, the greater the number of citations that can be found, regardless of its impact. To avoid this bias, we used yearly citations to better reflect the impact of the article. Third, the highly cited article only reflects the impact of the articles and the current focus of the field, but not the quality of the study. Thus, to fully evaluate a study field, information such as study design, rigor, or other measures need to be considered. Fourth, the rank of yearly citation counts might be affected by self-citations for some studies. However, the articles ranked after the top 50 most highly cited articles have limited yearly citation counts and do not affect our results. We will also try to avoid the effect of self-citation in our future studies. Last, the majority of highly cited articles in bibliometric studies are derived from overlapping lists of established journals indexed in major secondary databases. Some studies have reported that recently collected papers should have at least 2 years of historical accumulation of enough citation volume to establish bibliometric reliability (8). Therefore, our study can objectively reflect the hotspots and trends in all related studies up to 2017.

### Conclusion

This study provides an important and comprehensive analyzation of the most cited articles in the field of imaging application in children with CP over the past 29 years. The future direction of this field may lead to multi-modality studies with high-level evidence-based design that investigate the mechanism, prognosis, and therapeutic efficacy of CP. These studies recognized the important study progress in this field and provided a valuable framework and direction toward diagnosis, mechanism, treatment, and prognosis of CP. By offering important insights into the historical trends in this highly active and promising field, these results might have a strong impact on future research.

## Data Availability Statement

All datasets generated for this study are included in the article/supplementary material.

## Author Contributions

FW and XW performed the literature search, preliminary article analysis, drafted, and revised the paper. FW, HL, and JZ performed the literature search and screened articles. XW, MW, CL, ZZ, and XZ performed the literature search and download. FW, JY, XL, HJ, and TH analyzed the data and revised the draft manuscript. JY and HL oversaw the project and concept design, monitored the data collection, and revised the draft manuscript. All authors contributed substantially to the final article.

## Conflict of Interest

The authors declare that the research was conducted in the absence of any commercial or financial relationships that could be construed as a potential conflict of interest.
